# Roof-dependent Macro-reentrant Left Atrial Flutter Masked by Probable Epicardial Conduction via the Septopulmonary Bundle

**DOI:** 10.31662/jmaj.2025-0112

**Published:** 2025-06-20

**Authors:** Masataka Nakatsuka, Masao Takemoto, Togo Sakai, Takuya Tsuchihashi

**Affiliations:** 1 The Cardiovascular Center, Social Medical Corporation Steel Memorial Yawata Hospital, Kitakyushu, Japan

**Keywords:** ablation, atrial flutter, atrial tachycardia, case report, septopulmonary bundle

## Abstract

We encountered a 77-year-old woman who had a roof-dependent macro-reentrant left atrial (LA) flutter (LAFL), masked by probable epicardial conduction via the septopulmonary bundle (SPB). The SPB, an epicardial connection in the LA, may contribute to the maintenance and complexity of LAFL. Thus, when evaluating LAFL, physicians should consider the potential involvement of the SPB. Detailed observation using a three-dimensional mapping and comprehensive electrophysiological pacing maneuvers is crucial for identifying the re-entry circuit of LAFL.

## Introduction

Recent reports indicate that the septopulmonary bundle (SPB) may contribute to the maintenance and complexity of the left atrial (LA) flutter (LAFL), especially after the ablation of atrial fibrillation (AF) ^(1), (2)^. In contrast, we present a case of roof-dependent atrial flutter (AFL) masked by probable conduction via the SPB.

## Case Report

**Figure 1.**
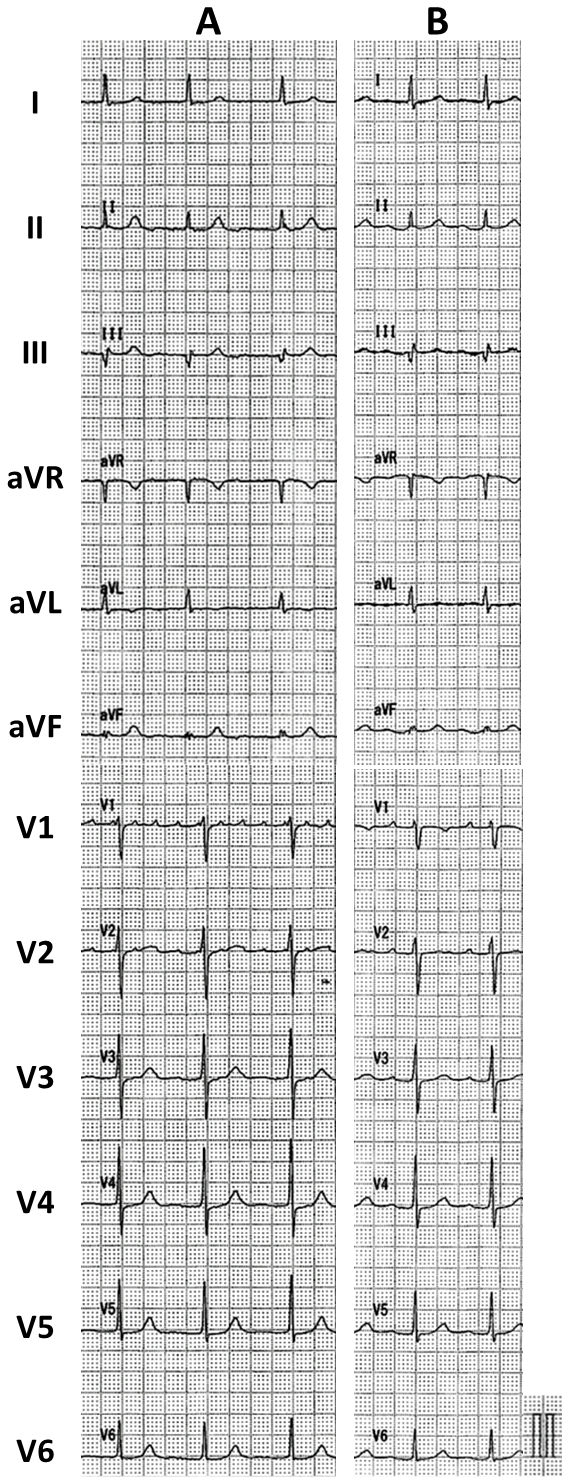
The 12-lead electrocardiogram on admission (A) and 2 years after ablation (B).

**Figure 2.**
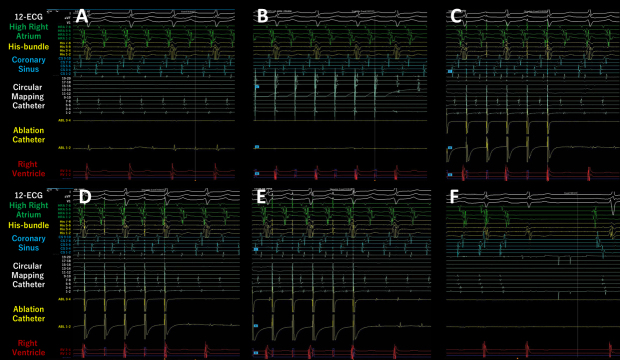
The intracardiac electrocardiograms during atrial tachycardia (AT)/atrial flutter (AFL) (A). Overdrive endocardial pacing using a circular mapping catheter or ablation catheter at the left atrial (LA) posterior wall (B), anterior wall (C), LA roof (D), and LA floor (E) resulted in acceleration of the AT/AFL to the pacing cycle length (CL) and concealed intracardiac entrainment (B-E). Post-pacing interval and CL at each site corresponded. Termination of AT/AFL during the delivery of radiofrequency energy (F).

**Figure 3.**
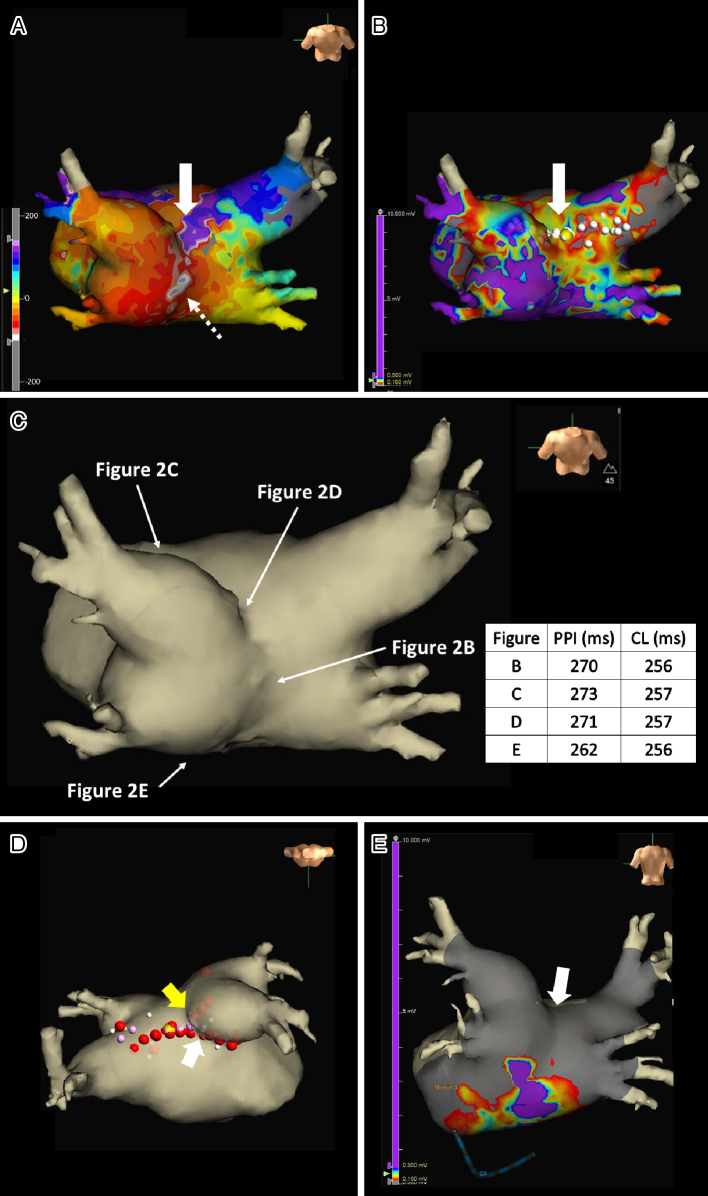
Local activation time isochronal (LATI) (A) and voltage (B) mapping images from an upper side view (A, B) during atrial tachycardia/atrial flutter. At first, this mapping (A) demonstrated that the earliest activation site was located within the left atrial (LA) posterior wall (LAPW) (white dotted arrow in A). However, careful observation revealed an ‘early meets late’ region on the roof and a slow conduction zone on the LA roof on the LATI map (white arrow in A), and a low voltage in this area on the voltage map (white arrow in B). Anatomy mapping image from a posteroanterior view (C). White arrows in C indicated the sites of the overdrive endocardial pacing. Radiofrequency energy was delivered (red dots in D) to both the LA roof line (yellow arrow in D) and the anterior line of the left superior pulmonary vein (PV) (white arrow in D). LATI mapping image after PVs and LAPW isolations from a posteroanterior view (E) during sinus rhythm using the EnSite system. CL: cycle length; PPI: post-pacing interval.

**Figure 4.**
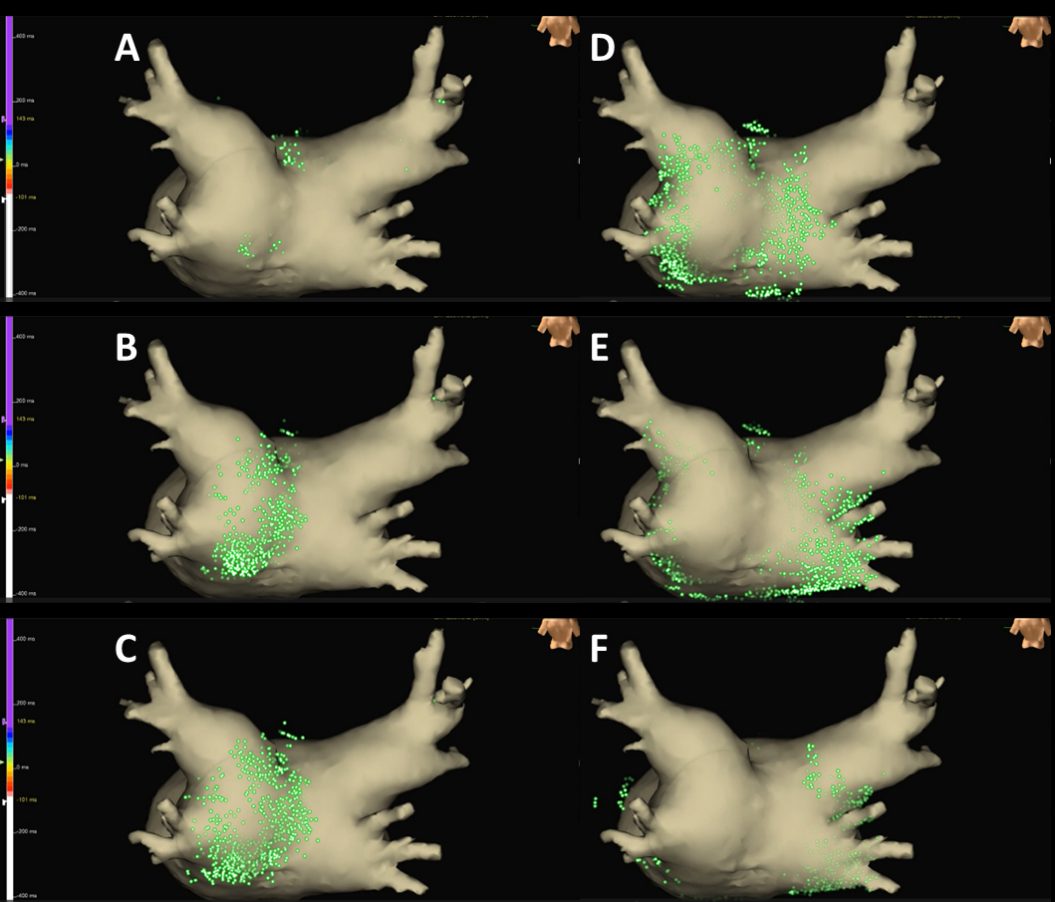
Sparkle mapping images of the posteroanterior region using the EnSite system during atrial tachycardia/flutter demonstrated that the earliest activation site was located within the LAPW, with the electrical signal spreading centrifugally from this site. AT: tachycardia; AFL: atrial flutter; CL: cycle length; LATI: local activation time isochronal; LA: left atrial; LAPW: left atrial posterior wall; PV: pulmonary vein; PPI: post-pacing interval.

Written consent was obtained from the patient to publish the information, including her photographs. A 77-year-old female, with a history of permanent pacemaker implantation for sick sinus syndrome (SSS) 3 years prior and no history of arrhythmia other than SSS or ablation before this event, was admitted for an ablation of intolerable symptomatic drug-refractory atrial tachycardia (AT)/AFL (Figure 1A). During AT/AFL, an intracardiac electrogram revealed a cycle length (CL) of 257 ms, with the earliest activation site at the distal coronary sinus (Figure 2A). Following a double transseptal puncture, local activation time isochronal and sparkle mapping were performed using a three-dimensional (3D) mapping system (EnSite™, Abbott) with a high-density mapping catheter (Advisor™ HD grid catheter, Abbott) during AT/AFL. Initially, this mapping demonstrated that the earliest activation site was located within the LA posterior wall (LAPW) (white dotted arrow in Figure 3A), with the electrical signal spreading centrifugally from this site on the EnSite™ sparkling map (Figure 4A-F). Since the observed epicardial conduction, likely via SPB, might play an important role in initiating and maintaining AT ^(3)^, it was hypothesized that the breakout site of this AT/AFL might be at the junction with the LAPW of SPB ^(1), (2), (4)^. However, careful observation revealed an “early meets late” region on the roof and a slow conduction zone on the LA roof (white arrow in Figure 3A) on the activation map, and a low voltage in this area on the voltage map (white arrow in Figure 3B)). Overdrive endocardial pacing with a CL of 240 ms using a circular mapping catheter (Optima™, Abbott) or ablation catheter (TactiCath SE™, Abbott) with an output of 10 V at the LAPW, right anterior wall, LA roof, and LA floor resulted in acceleration of the AT/AFL to the pacing CL and concealed intracardiac entrainment (Figure 2B-E). Post-pacing interval (PPI) and CL at each site corresponded (Figure 3C). Radiofrequency energy with an open irrigated ablation catheter was delivered to the roof of the slow conduction zone (yellow arrows in Figure 3A and B) rather than to the earliest activation site in the LAPW (white dotted arrow in Figure 3A) with a maximum power of 30 W, leading to steady termination of this AT/AFL (Figure 2F). This confirmed the diagnosis of a roof-dependent macro-reentrant LAFL masked by probable conduction via the SPB. Subsequently, to complete the roofline between the right and left superior (LS) pulmonary veins (PVs), intensive and precise radiofrequency energy was delivered to both the LA roof line (yellow arrow in Figure 3D) and anterior line of LSPV (white arrow in Figure 3D) and the endocardial breakthrough site of the SPB inside the LAPW ^(5)^. During the 30-minute observation period, programmed stimulation did not induce arrhythmias following isoproterenol administration.

Six months after the LAFL ablation, the patient returned because of AF. The patient subsequently underwent PV isolation (PVI) to create a line at the bottom of the LAPW (Figure 3E). The patient did not have any AFL, AF recurrence, or symptoms for 2 years after arrhythmia ablation (Figure 1B).

## Discussion

The SPB runs epicardially from the anterior interatrial raphe, ascends obliquely, and combines with longitudinal fibers from the anterior vestibule to pass over the dome between the left and right PVs on the LAPW ^(2)^. Recent reports indicate that this bundle may contribute to the complexity of the LAFL, especially after the ablation of AF ^(1), (2)^. In this case, the activation and propagation maps showed the earliest activation site within the LAPW (white dotted arrow in Figure 3A), with the electrical signal spreading centrifugally from this site (Figure 3A and 4A-F), indicative of a focal AT pattern. Since we experienced and reported that the SPB might play an important role in initiating and maintaining AT ^(3)^, we empirically speculated that SPB may be associated with AT/AFL. However, careful observation revealed an ‘early meets late’ region on the roof and a slow conduction zone on the LA roof (white arrow in Figure 3A) on the activation map, and a low voltage in this area on the voltage map (white arrow in Figure 3B). Thus, we hypothesized that this AT/AFL might be a roof-dependent macro-reentrant LAFL masked by probable conduction via the SPB. Endocardial pacing at the LAPW, right anterior wall, LA roof, and LA floor was then performed, resulting in the acceleration of the AT/AFL to the pacing CL and concealed intracardiac entrainment (Figure 2B-E) and PPI and CL at each site corresponded (Figure 3C), indicating a roof-dependent macro-reentrant AFL. Finally, radiofrequency energy was delivered to the roof of the slow conduction zone (white arrows in Figure 3A and B) rather than to the earliest activation site in the LAPW, which might be the junction of SPB ^(1), (2), (4)^, resulting in steady termination (Figure 2F). This led to a diagnosis of a roof-dependent macro-reentrant LAFL masked by probable conduction via the SPB. Therefore, when encountering an LAFL, physicians should consider the possibility of SPB involvement as it may contribute to its complexity. Detailed observations using 3D mapping and comprehensive electrophysiological pacing maneuvers are crucial for identifying the re-entry circuit of the AFL.

The LA roof myocardium is thicker because of the presence of the SPB between the superior PVs ^(6)^. Consequently, the roof lines are often incomplete due to this complex anatomy involving the SPB ^(4)^, and achieving block typically requires isolation of the posterior wall ^(5)^. Therefore, in this case, to complete the roofline between the right and LSPV and prevent SPB-dependent AFL, intensive and precise radiofrequency energy was delivered to both the LA roof line (yellow arrow in Figure 3D) and anterior line of LSPV (white arrow in Figure 3D) and the endocardial breakthrough site of the SPB inside the LAPW ^(5)^. In the second session, 3D mapping and pacing within the LAPW isolation lines after PVI and LAPW bottom line creation showed complete entrance and exit blocks (Figure 3E). These findings suggest that the complete abolition of epicardial conduction via the SPB was achieved in the first session (white arrow in Figure 3E).

AFL ^(7)^ and SSS ^(8)^ transition to and complicate AF. In this case, AF developed 6 months after de novo LAFL ablation. Thus, in the future, the treatment approach might be improved by treating AF, including PVI and LAPW isolation, during the first session. However, since this is a case report on a single patient, larger randomized clinical trials, including patients with LAFL, should be conducted to determine the generalizability of these results.

## Article Information

### Conflicts of Interest

None

### Acknowledgement

We thank Asami Yamada, Kensuke Kawasaki, Tomomi Hatae, Kyohei Shimamura, Tsutomu Yoshinaga, and Shu Takata, for their technical assistance with the electrophysiological study in the cardiac catheterization laboratory.

### Author Contributions

All doctors were involved in case management. Masataka Nakatsuka, Togo Sakai, and Masao Takemoto performed the ablation and wrote this manuscript.

### Approval by Institutional Review Board (IRB)

IRB Approval Code

24-56.

Name of the Institution

This study was approved by the Institutional Review Committee and Ethics Review Board of our hospital and the Ethical Review Board of the Steel Memorial Yawata Hospital.

### Informed Consent

Written informed consent was obtained from the patient.
